# Nanocomposites from β-Pinene and α-Pinene Copolymer: Synthesis, Characterization, and Antioxidant Evaluation

**DOI:** 10.3390/polym17172378

**Published:** 2025-08-31

**Authors:** Hodhaifa Derdar, Zakaria Cherifi, Geoffrey Robert Mitchell, Artur Mateus, Meziane Zerrouki, Naima Hammoudi, Khaldoun Bachari, Redouane Chebout, Fouzia Touahra, Abdelghani Bouchama, Amine Harrane, Rachid Meghabar

**Affiliations:** 1 Centre de Recherche Scientifique et Technique en Analyses Physico-Chimiques (CRAPC), BP 10 384, Siègeex-Pasna Zone Industrielle, Bou-Ismail 42004, Algeria; zakaria.cherifi.17@gmail.com (Z.C.); k.bachari@mesrs.dz (K.B.); redouane.chebout@crapc.dz (R.C.); ftouahra@gmail.com (F.T.); el3atik@hotmail.fr (A.B.); 2 Laboratoire de Chimie des Polymères (LCP), Département de Chimie, Faculty of Exact and Applied Sciences, Oran 1 University Ahmed Benbella, BP N° 1524 ElM’Naouar, Oran 31000, Algeria; amine.harrane@univ-mosta.dz (A.H.); meghabar.rachid@univ-oran1.dz (R.M.); 3 Centre for Rapid and Sustainable Product Development, Institute Polytechnic of Leiria, 2430-080 Marinha Grande, Portugal; 4 ISISE—Institute for Sustainability and Innovation in Structural Engineering, Department of Civil Engineering, University of Coimbra, 3004-531 Coimbra, Portugal; artur.mateus@dec.uc.pt; 5 Department of Chemistry, FSEI University of Abdelhamid Ibn Badis—Mostaganem, Mostaganem 27000, Algeria; zerouki.meziane.etu@univ-mosta.dz (M.Z.); hamoudi.naima@live.fr (N.H.); 6 Plateau Technique d’Analyses Physico-Chimiques, PTAPC-Mostaganem, Mostaganem 27000, Algeria

**Keywords:** copolymerization, nanocomposites, α- and β-pinene, nano-clay, antioxidant activity

## Abstract

In this study, we present a novel and straightforward approach for the synthesis of copolymers and nanocomposites based on α- and β-pinene, employing an eco-friendly and cost-effective nano-reinforcing filler. The copolymers (α-co-β-P) were produced through cationic copolymerization, using AlCl_3_ as a catalyst. The structural characterization of the resulting copolymer was validated through FT-IR, ^1^H-NMR spectroscopy, and differential scanning calorimetry (DSC). The molecular weight of the obtained polymer is determined by Gel Permeation Chromatography (GPC) analysis and is about 4500 g/mol. Nanocomposites (α-co-β-P/Clay 2, 5, 8, and 10% by weight of nano-clay) were synthesized by combining clay and α-co-β-P copolymer in solution using ultrasonic irradiation. This ultrasound-assisted method was employed to enhance and assess the structural, morphological, and thermal properties of the pure copolymer. The morphology of the resultant nanocomposites was characterized using infrared spectroscopy (FT-IR), X-ray diffraction (XRD), scanning electron microscopy (SEM) and transmission electron microscopy (TEM). Thermogravimetric analysis (TGA) revealed that the nanocomposites exhibit a higher degradation temperature compared to the pure copolymer. The analyses provided evidence of the chemical modification of nano-clay layers and their uniform dispersion in the α-co-β-P copolymer matrix. Exfoliated structures were achieved for lower clay concentration (2% by weight), while intercalated structures and immiscible regions were observed for higher clay concentrations (5, 8, and 10% by weight). The antioxidant activity of α-pinene, β-pinene, and the obtained nanocomposites were studied using DPPH (2,2-diphenyl-1-picrylhydrazyl) as a model free-radical. The results demonstrate a significant antioxidant potential of the nanocomposites, showcasing their ability to effectively neutralize free-radicals. Finally, a novel procedure was devised for the rapid synthesis of copolymers and nanocomposites using α- and β-pinene.

## 1. Introduction

The creation of polymers from renewable monomers is now a major focus of numerous current research initiatives worldwide. The bulk of study has been on polymers derived from renewable resources, out of all the different types of polymers that have been examined [1,2,3]. Terpenes’ reactivity in organic chemistry synthesis has been extensively studied [4], but their application in polymer science is still restricted. Monocyclic terpenes that are frequently present in citrus and pin peel essential oils are very useful in polymerization processes because of their allylic structure (CH_2_=CH-CH_2_Y) and the presence of double bonds that provide them with the required bifunctionality for polymerization [5,6]. Reviews of the literature show that chemists have been investigating alternatives to poly-terpenes derived from petroleum-based distillates [7]. Since the majority of terpenes cannot be homopolymerized because of things like bulk steric hindrance [8], low stabilization energy between monomers, and the transition state radicals [9], no workable alternatives have been created up to this point. The exceptions to this rule include limonene, α-pinene, and β-pinene, which have been effectively polymerized with catalysts such as Friedel–Crafts, Ziegler–Natta [10,11,12], and clay [13,14].

One of the main ingredients in natural turpentine, β-pinene, is utilized commercially to make resins for a variety of uses [15]. It is a reactive monomer that creates polymers with moderate molecular weights (Mn~2000) through cationic isomerization polymerization [16]. Because of its special qualities, β-pinene can be copolymerized with limonene to create flexible polymers with low glass transition temperatures (Tgs). These copolymers are widely used as additives in pharmaceuticals, agricultural chemicals, and the food sector, as well as in the creation of tastes and scents for cosmetics and perfumes [17,18,19]. Additionally, they are often used in the synthesis of fine compounds [20]. The tricyclic monoterpene β-pinene has an isomer called α -pinene, which is used to treat excessive bronchial discharge and is well-known for its antibacterial qualities. It is a component of turpentine oil and can be found in a variety of plants, including ginger, sage, lavender, and mint. With the exception of the oxygen atom, the α-pinene molecule is quite similar to camphor. Although pine oil is found naturally, it can also be made by reacting α-pinene with an acid to create α-terpineol (C_10_H_18_O), the primary ingredient in pine oil that gives juniper berries their distinctive aroma [21].

Nanocomposites, a novel class of materials reinforced with nanoscale particles, have attracted increasing attention in recent decades. In the early 1990s, Toyota researchers were among the first to investigate these novel materials. By adding clays to polyamide-6 by in situ polymerization, they reportedly showed a notable improvement in dimensional stability [22]. Numerous scientific domains now have new prospects in polymer matrix nanocomposites as a result of these discoveries [23]. Eco-friendly materials are increasingly being used in place of hazardous polymer-based nanocomposites in recent years. Using a polymer matrix and adding the right quantity of clay as a reinforcing agent greatly improves the physicochemical characteristics of these nanocomposites [24,25]. Depending on the degree of contact between the modified polymer and the clay, two primary types of nanocomposite structures can form: intercalated and exfoliated. Numerous procedures, including solution blending of polymers and in situ polymerization, can be used to create these nanocomposites [26].

Our survey of the literature indicates that there is hardly any research on the use of AlCl_3_ as a catalyst in the synthesis of α- and β-pinene copolymers and the antioxidant activity of nanocomposites/α- and β-pinene-based copolymers using DPPH (2,2-diphenyl-1-picrylhydrazyl). With the use of an ultrasound-assisted technique, the main goals of this study are to examine the catalytic characteristics of AlCl_3_ as a novel catalyst for the copolymerization of α-pinene and β-pinene and to investigate nano-clay as a novel nano-reinforcing filler for the creation of an antioxidant nanocomposites/poly-terpenes based copolymer. In our earlier research, we emphasized the benefits of using this kind of nano-reinforcing filler in different nanocomposite synthesis, showing that it can improve the mechanical and thermal characteristics of the resultant copolymers [27,28,29,30,31].

## 2. Materials and Methods

### 2.1. Materials

Aluminum chloride (AlCl_3,_ 98%), Toluene (99.8%), methanol (CH_3_OH, 99.9%), dichloromethane (CH_2_Cl_2_, 99.8%), α-pinene (97%), β-pinene (97%), NaOH, HCl, MgSO_4_, and nano-clay were all purchased from Sigma-Aldrich (St. Louis, MO, USA) and used as received without further purification. The antioxidant study employed 2,2-Diphenyl-1-picrylhydrazyl (DPPH), which was also acquired from Sigma-Aldrich. The nanocomposites were prepared using an ultrasonic device consisting of a jacketed glass tank equipped with an ultrasonic horn (13.6 mm diameter, non-replaceable tip made of titanium alloy Ti-6Al-4V) connected to a Sonics VC-750 Vibra Cell generator (Sonics & Materials, Inc., Newtown, CT, USA).

### 2.2. Copolymerization Procedure

**Figure 1:**
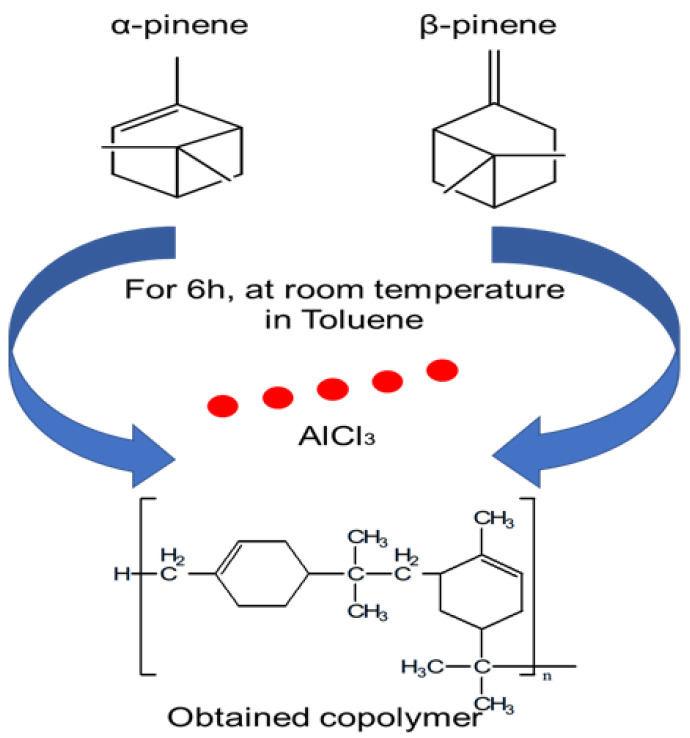
Copolymerization reaction of α-pinene and β-pinene using Toluene at room temperature and AlCl_3_ as catalyst.

α-pinene and β-pinene were copolymerized in solution for 6 hours at room temperature with AlCl_3_ as a catalyst. As shown in Figure 1, 10 mL of Toluene was mixed with 0.03 mol of α-pinene and β-pinene and 5% by weight of AlCl_3_. When AlCl_3_ was added, the solution became orange, signifying the start of the copolymerization process. As the polymerization process progressed, the fluid became darker and thicker. A total of 10 mL of HCl (0.1 M) was added to the reaction mixture and stirred until the orange color disappeared in order to extract AlCl_3_. Following that, distilled water and NaOH (0.1 M) were used to repeatedly clear the organic phase. Magnesium sulfate (MgSO_4_) was used to dry the organic phase, and the Toluene was then evaporated. The products underwent an overnight vacuum drying process after being dissolved in THF and precipitated in cold methanol (MeOH).

### 2.3. Elaboration of Nanocomposites Copolymer/Clay (α-co-β-P)/Clay)

**Figure 2:**
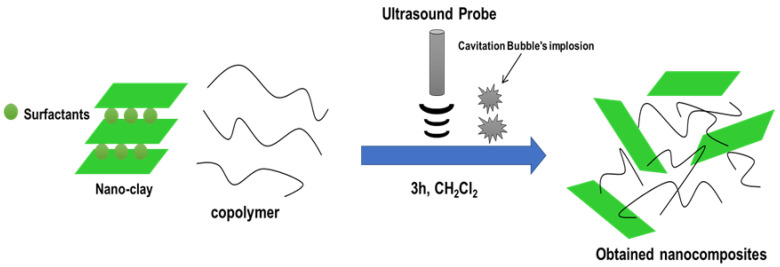
Elaboration procedure of nanocomposites using ultrasonic irradiation for 3 h and CH_2_Cl_2_ as solvent.

α-co-β-P/clay nanocomposites were created by combining clay with polymers in a solution (see Figure 2). The copolymer was produced by dissolving 0.5 g in 20 mL of dichloromethane (CH_2_Cl_2_). Stirring the liquid for ten more minutes allowed the copolymer to dissolve entirely. After adding 2% by weight of nano-clay, the solution was treated for three hours using an ultrasonic-assisted approach [32]. After filtering and precipitating in methanol (MeOH), the resultant nanocomposite was vacuum-dried for the entire night. For nano-clay additions of 5%, 8%, and 10% by weight to α-co-β-P the identical process was carried out again. α-co-β-P/clay was the name given to the samples (see to Table 1 for experimental circumstances).

### 2.4. Characterization

The structure of the obtained copolymer was confirmed by ^1^H-NMR spectra, recorded on a Bruker-Avance 400 MHz (CRAPC, Algeria) apparatus using deuterated chloroform (CDCl_3_) as the solvent. Infrared spectroscopy (FT-IR) was used to examine the functional groups of the copolymer, nano-clay, and nanocomposites using a BRUKER Optics Diamond-ATR (Invenio R), CRAPC, Algeria. Differential scanning calorimetry (DSC) was used to examine the thermal characteristics of the α-co-β-P copolymer using a NETZSCH DSC 204 F1 calorimeter at CDRSP, Leiria, Portugal. The experiments were carried out in an inert atmosphere with a flow rate of 50 mL/min, heating the mixture from room temperature to 500 °C at a rate of 20 °C/min. The molecular weight distribution of the obtained polymer was analyzed using a GPC-PL120 apparatus equipped with a refractive index detector. The analysis was performed at 27.5 °C using dichloromethane (CH_2_Cl_2_) as the mobile phase at a flow rate of 1.0 mL/min. Calibration was carried out using a series of polystyrene standards with a narrow molecular weight distribution, covering a range from approximately 500 to 100,000 g/mol. The molecular weights of the samples were calculated relative to these standards. Using a Bruker AXS D8 diffractometer (Cu-Kα radiation) from Oran 1 university, Algeria, the morphology of the modified clay and the produced nanocomposites was investigated using X-ray diffraction (XRD) patterns and field-emission scanning electron microscopy (FEG-SEM) using an electron microscope made by Thermo Fisher (Apreo 2C) at CRAPC, Algeria. Transmission electron micrographs were performed using a Hitachi 8100 at Lisbon University, Portugal. To investigate the thermal stability of the nanocomposites, thermogravimetric analysis (TGA) was performed on a PerkinElmer STA 6000 at CDRSP, Leiria, Portugal, under nitrogen at temperatures between 30 and 700 °C at a heating rate of 20 °C/min. The Agilent (Cary 60) UV–Visible spectrometer at CRAPC, Algeria, was used to investigate the antioxydant activity.

## 3. Results and Discussion

### 3.1. Characterization of the Obtained Copolymer (α-co-β-P)

#### 3.1.1. ^1^H-NMR Measurements

**Figure 3:**
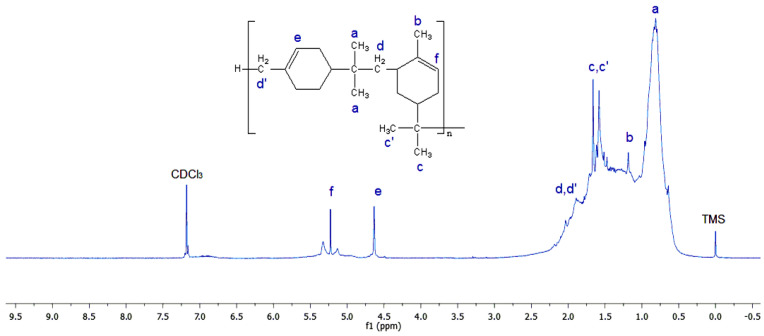
^1^H-NMR (Bruker-Avance 400 MHz) spectrum of the obtained copolymer (α-co-β-P) using CDCl_3_.

Figure 3 displays the ^1^H-NMR spectra of the obtained copolymer. The proposed structure was verified and investigated further using the ^1^H-NMR spectra. A signal at 0.9 ppm and other peaks that represent the protons of the methyl group are clearly visible in the ^1^H-NMR spectra of (α-co-β-P). The peak (b) at 1.2 ppm in the spectra of the resulting copolymer is caused by the protons of the methylene group (-CH2). The characteristic resonance peaks (e and f) between 4.7 and 5.4 ppm correspond to the protons implicated in the internal double bonds (-CH=CH-) of α- and β-pinene. The comparison of the ^1^H-NMR spectra of the obtained copolymer with those of the monomers β-pinene [33] and α-pinene [34] confirms the success of the copolymerization reaction using AlCl_3_ as a catalyst.

#### 3.1.2. FT-IR Measurements

**Figure 4:**
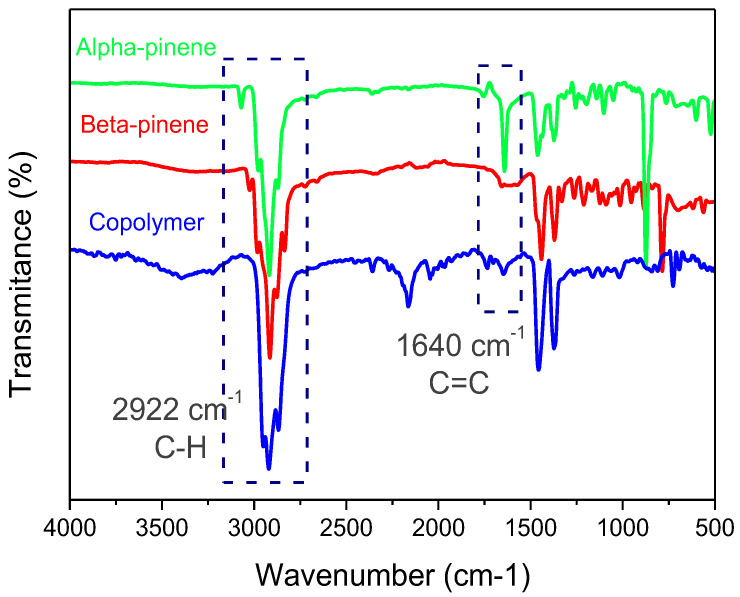
FT-IR spectra of α-pinene, β-pinene, and the obtained copolymer.

FT-IR measurements were also performed to confirm the copolymer structure. Figure 4 shows the FT-IR spectra of α-pinene, β-pinene, and the synthesized copolymer. The copolymer spectrum exhibits the disappearance of double-bond peaks previously associated with the absorption of β-pinene at 1217 and 956 cm^−^^1^ [35], which validates the copolymerization process. However, the band assigned to C=C stretching vibration at 1644 cm^−^^1^, visible in both monomers, is decreased in the spectrum of the copolymer. In addition, the FT-IR spectrum of the synthesized copolymer exhibits an intense absorption band at 2922 cm^−^^1^, which is attributed to the asymmetric stretching vibration of the C-H bonds in methylene (-CH_2_-) groups. This prominent band reflects the presence of aliphatic chains within the polymer structure, indicating successful incorporation of both monomers. The disappearance or significant reduction in the characteristic bands corresponding to the C=C stretching vibrations of the monomers (typically observed around 1640–1670 cm^−1^) further supports the occurrence of the polymerization reaction. These findings are in good agreement with the ^1^H-NMR analysis, which shows the disappearance of the olefinic proton signals of α-pinene and β-pinene. Therefore, the combined FT-IR and NMR results provide strong and complementary evidence for the successful synthesis of the (α-co-β-P) copolymer.

#### 3.1.3. Thermal Study of the Obtained Copolymer with DSC

**Figure 5:**
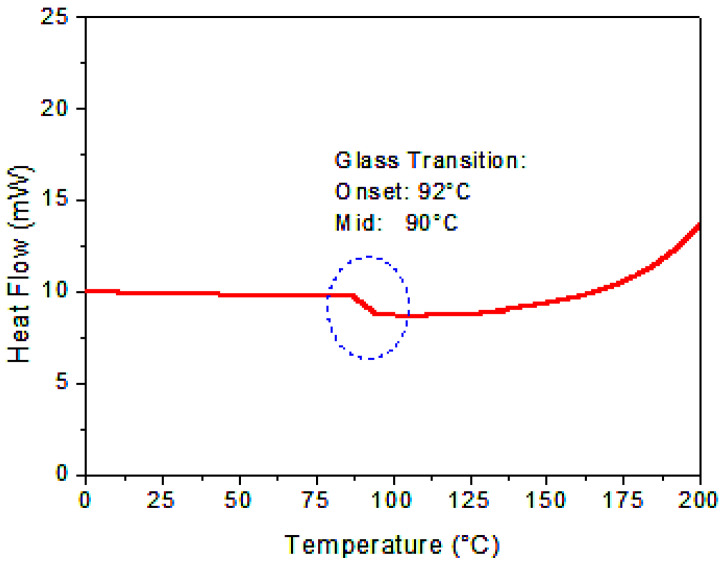
DSC curve at 20 °C/min of the obtained copolymer.

Using differential scanning calorimetry (DSC), the produced copolymer’s thermal characteristics were assessed. Figure 5 displays the α-co-β-P copolymer’s DSC thermogram. The copolymer’s glass transition temperature (Tg) was found to be at the interval of 90–95 °C based on DSC curve analysis. As previously mentioned in the literature, this Tg value is halfway between the stated Tg values of poly(α-pinene) and poly(β-pinene) [36,37]. The successful copolymerization of α-pinene with β-pinene is strongly supported by the change in Tg relative to the respective homopolymers, which also confirms that both monomer units are integrated into the same polymer structure. Additionally, it seems that using AlCl_3_ as a catalyst successfully encourages the copolymerization of α-pinene with β-pinene. These thermal analysis results are consistent with the structural characterization obtained by FT-IR and NMR analysis.

#### 3.1.4. GPC Measurements

**Figure 6:**
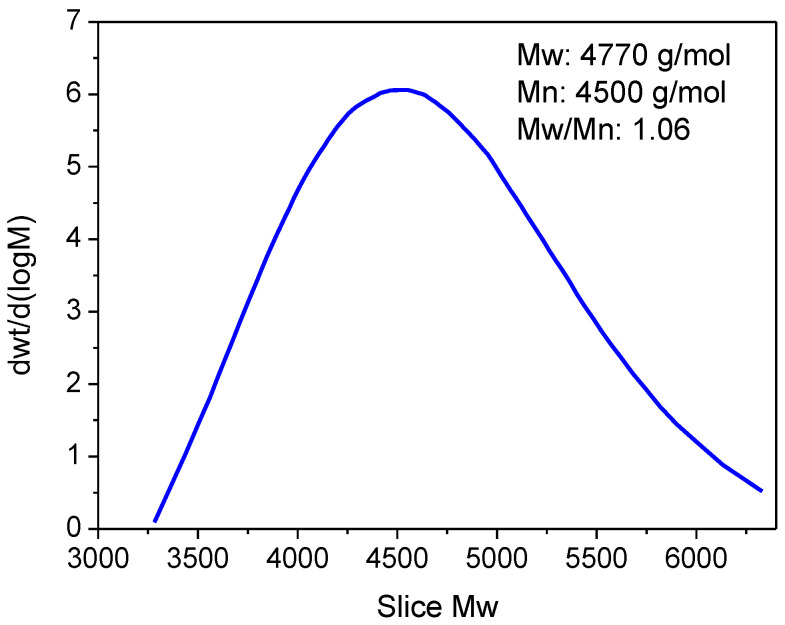
GPC chromatogram of the obtained copolymer.

Gel Permeation Chromatography (GPC) was used to determine the molecular weight distribution of the synthesized copolymer: the results are displayed in Figure 6. According to the GPC chromatogram, the copolymer’s number-average molecular weight (Mn) is about 4500 g/mol, and its weight-average molecular weight (Mw) is roughly 4770 g/mol. This results in a polydispersity index (Mw/Mn) of 1.06. These findings suggest good control over the polymerization process because they show a relatively narrow molecular weight distribution. Even though the molecular weight is moderate when compared to similar copolymers produced through conventional radical polymerization [38], the low cost, strong catalytic activity, and ability to operate under mild conditions make AlCl_3_ a desirable catalyst for polymerization reactions. Its high reactivity toward natural monomers such as β-pinene and α-pinene, along with its ease of handling and compatibility with various solvents, further support its use in the synthesis of sustainable polymers.

### 3.2. Characterization of the Obtained Nanocomposites (α-co-β-P/Clay)

#### 3.2.1. FT-IR Analysis

**Figure 7:**
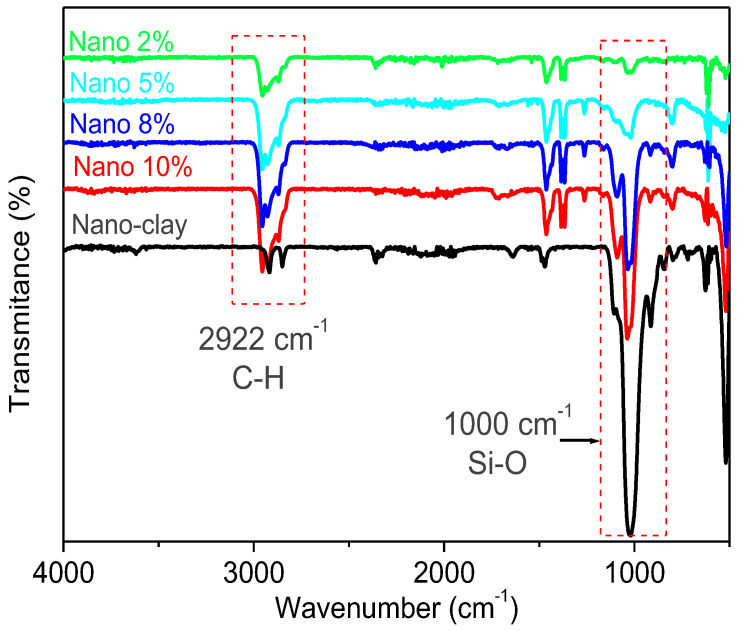
FT-IR spectra of the nano-clay, pure copolymer, and the obtained nanocomposites (2, 5, 8, and 10%).

The FT-IR spectra of the synthesized nanocomposites (α-co-β-P/clay 2%, 5%, 8%, and 10%) are shown in Figure 7. The vibration bands of the nanocomposites are seen to be quite similar to those of the nano-clay and to the structure of the pure copolymer. The copolymer’s C-H bonds are visible in the nanocomposites FT-IR spectra at 2922 and 2867 cm^−1^, which correspond to the methyl and methylene groups’ vibrations. Furthermore, the FT-IR spectrum of the pure copolymer does not exhibit any significant absorption band around 1000 cm^−1^, which is typically associated with the Si-O stretching vibrations characteristic of nano-clay structures [39]. This band, however, clearly appears in the spectra of the obtained nanocomposites, confirming the successful incorporation of nano-clay within the copolymer matrix. The absence of this Si-O band in the pure copolymer spectrum indicates that the polymer itself does not contain silicate structures, and its presence in the nanocomposite spectra further supports the interaction and dispersion of the clay within the copolymer matrix.

#### 3.2.2. XRD Analysis

**Figure 8:**
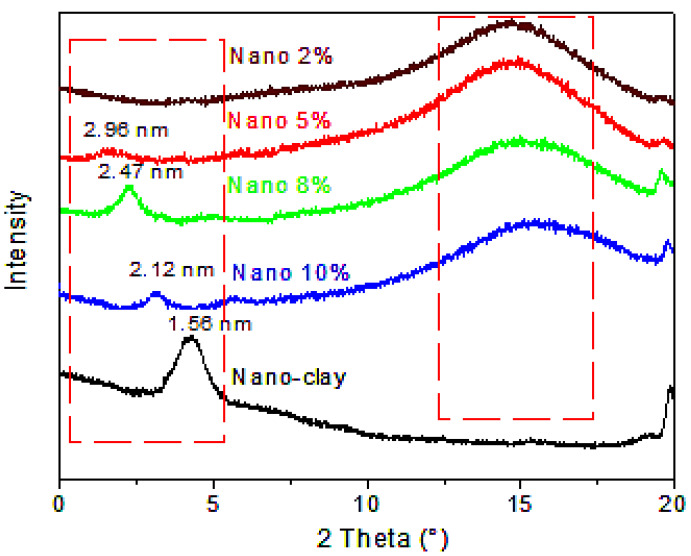
XRD patterns of the obtained nanocomposites (2, 5, 10, and 10%) and nano-clay.

The XRD patterns of the nano-clay and the nanocomposites are displayed in Figure 8. In the α-co-β-P/clay 2% sample, the distinctive basal diffraction signal of nano-clay about 2θ = 5° was entirely missing, suggesting that the clay had been exfoliated, allowing the α-co-β-P copolymer to diffuse effectively within the clay galleries. A single peak was seen for the nanocomposites containing 10%, 8%, and 5% nano-clay, which corresponded to interlayer distances of 2.12, 2.47, and 2.96 nm, respectively. These nanocomposites had interlayer distances that were almost double that of the nano-clay, which has an interlayer distance of 1.56 nm. The copolymer’s successful intercalation between the clay layers is confirmed by this outcome. These results are in line with what Kherroub et al. [40] found.

#### 3.2.3. SEM Analysis

**Figure 9:**
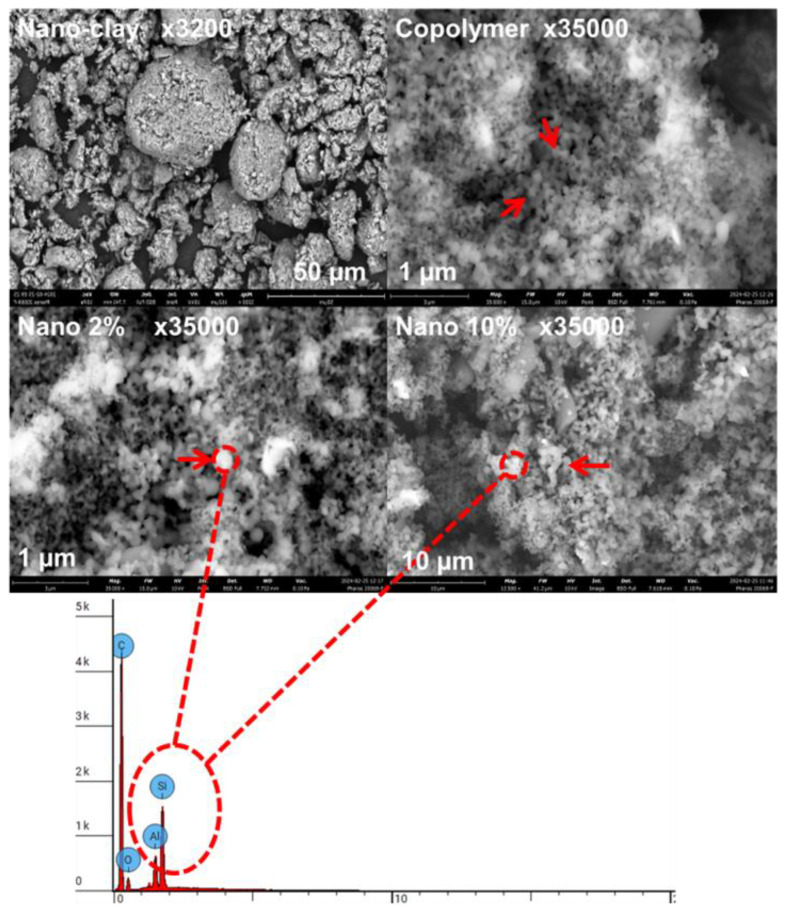
SEM images and EDS spectra of the obtained nanocomposites (2 and 10%) and nano-clay.

Figure 9 presents the SEM images of the synthesized nanocomposites (α-co-β-P/clay) prepared with 2% and 10% by weight of nano-clay, alongside the pure nano-clay. The morphology of the nano-clay shows well-defined layered structures typical of montmorillonite. In contrast, the nanocomposites, particularly at 10% by weight of clay, exhibit significant changes in morphology. At this amount, montmorillonite appears more organized and fragmented into smaller particle sizes, indicating a partial intercalation of the clay layers within the copolymer matrix. Examination of a 10 µm surface area of the nanocomposites reveals dispersed and loosely stacked montmorillonite plates, confirming good interaction with the copolymer. Additionally, for the nanocomposites containing 2% clay, the surface morphology becomes rougher and a continuous copolymer coating is observed over the clay surface, suggesting good compatibility of the clay within the copolymer matrix. These observations imply that at lower clay amounts the copolymer efficiently covers and interacts with the clay layers, while higher clay amounts favor more organized clay structures. Furthermore, energy dispersive X-ray spectroscopy (EDS) analysis was performed to confirm the elemental distribution and to demonstrate the successful dispersion of the clay within the copolymer matrix, showing characteristic peaks of silicon and aluminum associated with montmorillonite, thus supporting the morphological findings.

#### 3.2.4. TEM Analysis

**Figure 10:**
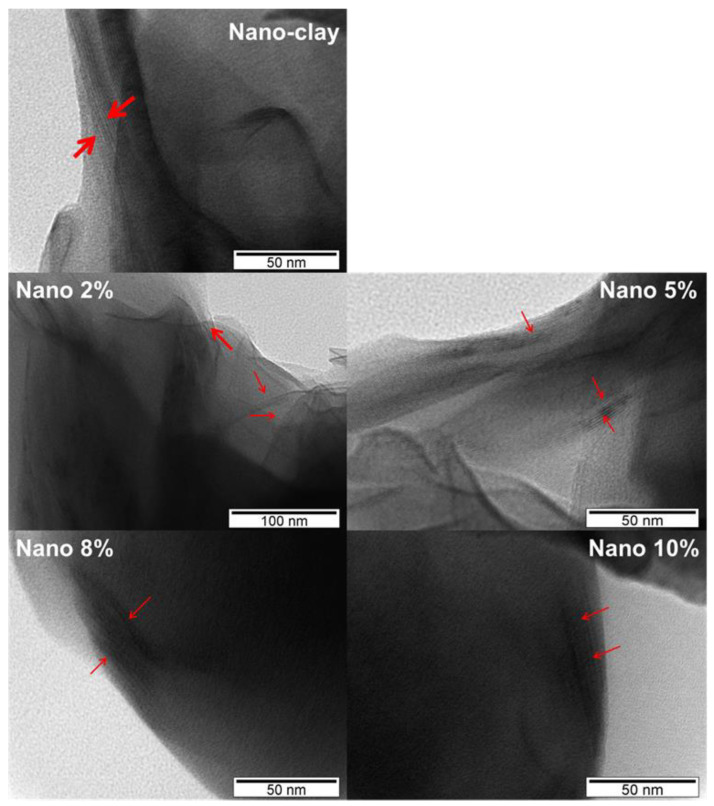
TEM images of the obtained nanocomposites (2, 5, 8, and 10%) and the nano-clay.

Figure 10 shows the transmission electron microscopy (TEM) images of nano-clay and the prepared nanocomposites with varying nano-clay amounts (2%, 5%, 8%, and 10% by weight). TEM analysis was employed to evaluate the dispersion of nano-clay within the copolymer matrix and to confirm the structural morphology suggested by the XRD results. In the image of nano-clay the layered silicate structure is clearly visible, appearing as dark, stacked platelets. In the nanocomposite containing 2% of nano-clay, the clay layers are relatively well-dispersed, with evidence of partial exfoliation. As the clay content increases to 5%, a similar partially intercalated morphology is observed, although a slight increase in agglomeration is noticeable. For the nanocomposites prepared with 8 and 10% by weight of nano-clay, the TEM images reveal more pronounced stacking and aggregation of silicate layers, indicating a tendency toward intercalated structures and reduced dispersion at higher filler loadings. These morphological changes suggest that lower nano-clay content favors better dispersion and exfoliation within the copolymer matrix, while higher loadings lead to structural reorganization and limited interlayer separation, consistent with the XRD findings.

#### 3.2.5. TGA

**Figure 11:**
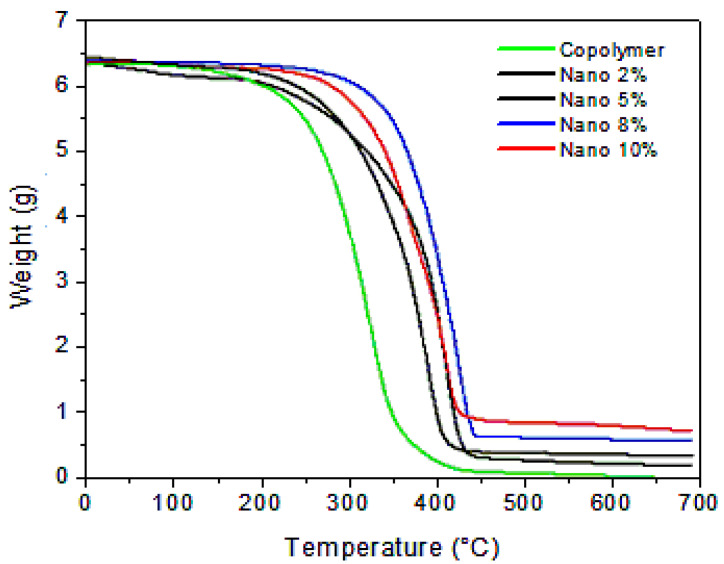
TGA curves of pure copolymer and nanocomposites (2, 5, 8, and 10%).

The TGA curves of the nanocomposites produced and the pure copolymer are shown in Figure 11, and Table 2 summarizes the key TGA data. It is clear that the pure copolymer and all nanocomposites experience a single-step weight loss. The thermal stability of the nanocomposites is improved with inclusion of nano-clay, as the TGA results suggest. The degradation temperature of the pure copolymer is 220 °C. However, the nanocomposites which contain 2, 5, 8, and 10% by weight of nano-clay exhibit significantly greater thermal stability with a degradation temperature of about 350 °C. TGA curves also show residual masses at 700 °C, with values exceeding 0.5 g. This behavior is attributed to the inorganic content of the nano-clay, which contains aluminum and other mineral components. These elements are thermally stable and do not decompose during the thermal degradation process.

The higher the copolymer content in the nanocomposite, the faster its degradation occurs. Prior work [41] indicates that the reasons for increased thermal stability are the presence of a carbonized protective layer. This layer is due to the fine dispersion of intercalated or exfoliated clay particles that serve as an inorganic barrier [42]. As a rule, the addition of exfoliated lamellar silicates increases the thermal stability of the polymers and their performance at elevated temperatures [43].

### 3.3. Antioxidant Activity

At a concentration of 0.004% *w*/*v*, a freshly manufactured solution of the 2,2-diphenyl-1-picrylhydrazyl (DPPH) radical in CH_2_Cl_2_ was prepared and kept in a dark place. Additionally, a solution containing the test chemical was made [44]. The experiment was started by mixing 2.5 mL of DPPH solution with 100 μL of each sample’s solution, then letting it sit in the dark for 30 min. At 515 nm, the absorbance was measured with a spectrophotometer. The equation used for calculating the DPPH radical scavenging capacity is as follows:

Free-radical scavenging activity (%) = ((Abs _DPPH_ − Abs _sample_)/Abs _DPPH_) ×100

where ABS _sample_ and Abs _DPPH_ are the absorbance of DPPH in sample solution and blank, respectively.

The 50% inhibitory concentration (IC50), the concentration required to inhibit the DPPH radical by 50%, was estimated from graphic plots of the dose–response curve.

**Figure 12:**
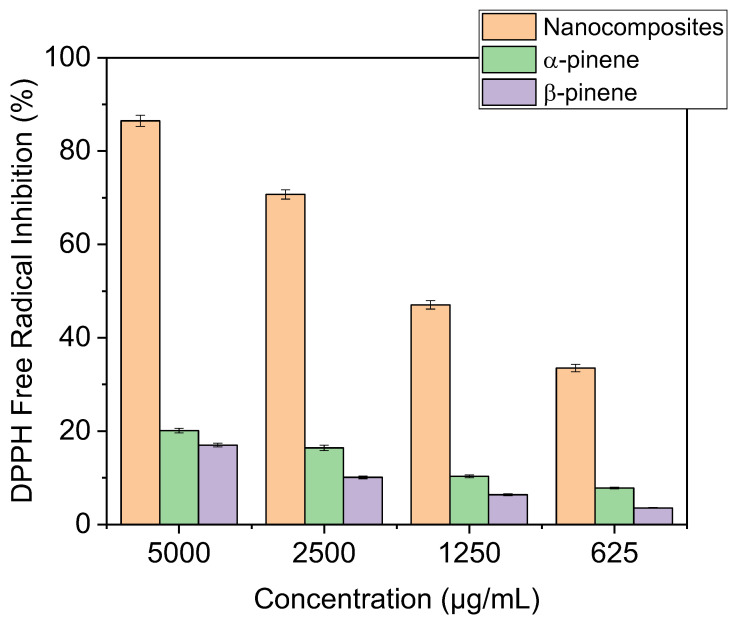
Free-radical scavenging activity of α-pinene, β-pinene and the obtained nanocomposites using DPPH at 0.004%w/v in CH_2_Cl_2_.

The antioxidant activities of α-pinene, β-pinene, and the synthesized nanocomposites (α-co-β-P/clay) were evaluated using the DPPH free-radical scavenging assay. As illustrated in Figure 12, both α-pinene and β-pinene exhibited relatively low antioxidant activity, with inhibition amounts below 20% even at high concentrations (5000 µg/mL) [45]. In contrast, the nanocomposites demonstrated a significantly enhanced antioxidant response. The highest DPPH radical inhibition recorded for the nanocomposites was 86.48%, corresponding to an IC_50_ value of 163.41 ± 1.03 µg/mL, indicating strong radical scavenging capacity. This notable improvement is likely due to the synergistic effect between the copolymer matrix and the nano-clay reinforcement, which may enhance electron transfer and the stabilization of DPPH radicals and promote radical scavenging via other mechanisms such as electron donation from allylic or conjugated systems [46,47,48], as confirmed by FTIR. 

In the study by Omnia et al. [49] on AN-co-St copolymers an increase in antioxidant activity was observed, with an IC_50_ value of 237.39 ± 3.87 μg/mL compared to our obtained nanocomposites based on poly-terpenes, indicating better antioxidant activity. This enhancement can be attributed to the introduction of new nanocomposites.

## 4. Conclusions

The influence of nano-clay, which was prepared and applied at varying ratios, on the properties of the α-co-β-P/clay nanocomposites was studied. XRD results indicated that the nanocomposite, containing 2% by weight of clay, was exfoliated, and the rest of the 5%, 8%, and 10% clay-containing nanocomposites were intercalated, thus resulting in an increase in the interlayer spacing. The thermogravimetric analysis further showed that the nanocomposites have better thermal stability than the pure copolymer, with an onset degradation temperature of about 350 °C. These improvements are a result of the interaction with the organic compounds of the modified clay and the copolymer chain. The clay’s reinforcing effect on the copolymer was evidenced by the increase in the rigidity of the system. Moreover, the SEM of the nanocomposites demonstrated certain particles being organized while other particles show disintegration into plates of montmorillonite layers, which confirmed the partial or full exfoliation of the montmorillonite that was incorporated into the copolymer matrix to prepare the nanocomposites. Additionally, these results were confirmed by TEM analysis. Furthermore, the antioxidant activity of the nanocomposites, evaluated using DPPH (2,2-diphenyl-1-picrylhydrazyl) as a model free-radical, demonstrated a significant antioxidant potential, highlighting the nanocomposites’ ability to effectively neutralize free-radicals. In addition, the study showed that it is possible to prepare such nanocomposites using the nano-clay as a nano-reinforcing filler.

## Figures and Tables

**Table 1:** Experimental conditions for the preparation of nanocomposites α-co-β-P/clay.

Samples	α-co-β-P	Nano-Clay	Time	Frequency	Yield
α-co-β-P/clay 2%	0.5 g	2% (wt)	3 h	20 KHz	100%
α-co-β-P/clay 5%	0.5 g	5% (wt)	3 h	20 KHz	100%
α-co-β-P/clay 8%	0.5 g	8% (wt)	3 h	20 KHz	100%
α-co-β-P/clay 8%	0.5 g	10% (wt)	3 h	20 KHz	100%

**Table 2:** Thermal stability parameters of copolymer and nanocomposites with different nano-clay amounts, from TGA curves.

Samples	Onset Degradation Tem °C	Max Degradation Tem °C	Residue at 700 °C (g)
Copolymer	220	380	0
Nanocomposites 2%	300	430	0.2
Nanocomposites 5%	310	420	0.5
Nanocomposites 8%	350	450	0.6
Nanocomposites 10%	330	470	0.9

## Data Availability

Data are contained within the article.

## Abbreviations

The following abbreviations are used in this manuscript:

α-co-β-P	α- and β-pinene based copolymer
α-co-β-P/clay	α- and β-pinene copolymer-based nanocomposites
DPPH	2,2-Diphenyl-1-picrylhydrazyl
